# Magnetoelastic interactions and magnetic damping in Co_2_Fe_0.4_Mn_0.6_Si and Co_2_FeGa_0.5_Ge_0.5_ Heusler alloys thin films for spintronic applications

**DOI:** 10.1038/s41598-021-87205-y

**Published:** 2021-04-07

**Authors:** O. M. Chumak, A. Pacewicz, A. Lynnyk, B. Salski, T. Yamamoto, T. Seki, J. Z. Domagala, H. Głowiński, K. Takanashi, L. T. Baczewski, H. Szymczak, A. Nabiałek

**Affiliations:** 1 Institute of Physics, Polish Academy of Sciences, Al. Lotników 32/46, 02-668 Warsaw, Poland; 2 Institute of Radioelectronics and Multimedia Technology, Warsaw University of Technology, Nowowiejska 15/19, 00-665 Warsaw, Poland; 3 Institute for Materials Research, Tohoku University, Sendai, 980-8577 Japan; 4 Center for Spintronics Research Network, Tohoku University, Sendai, 980-8577 Japan; 5 Institute of Molecular Physics, Polish Academy of Sciences, M. Smoluchowskiego 17, 60-179 Poznań, Poland; 6 Center for Science and Innovation in Spintronics, Core Research Cluster, Tohoku University, Sendai, 980-8577 Japan

**Keywords:** Magnetic properties and materials, Surfaces, interfaces and thin films, Magnetic properties and materials

## Abstract

Co_2_Fe_0.4_Mn_0.6_Si (CFMS) and Co_2_FeGa_0.5_Ge_0.5_ (CFGG) Heusler alloys are among the most promising thin film materials for spintronic devices due to a high spin polarization, low magnetic damping and giant/tunneling magnetoresistance ratios. Despite numerous investigations of Heusler alloys magnetic properties performed up to now, magnetoelastic effects in these materials remain not fully understood; due to quite rare studies of correlations between magnetoelastic and other magnetic properties, such as magnetic dissipation or magnetic anisotropy. In this research we have investigated epitaxial CFMS and CFGG Heusler alloys thin films of thickness in the range of 15–50 nm. We have determined the magnetoelastic tensor components and magnetic damping parameters as a function of the magnetic layer thickness. Magnetic damping measurements revealed the existence of non-Gilbert dissipation related contributions, including two-magnon scattering and spin pumping phenomena. Magnetoelastic constant B_11_ values and the effective magnetic damping parameter α_*eff*_ values were found to be in the range of − 6 to 30 × 10^6^ erg/cm^3^ and between 1 and 12 × 10^–3^, respectively. The values of saturation magnetostriction λ_S_ for CFMS Heusler alloy thin films were also obtained using the strain modulated ferromagnetic resonance technique. The correlation between α_*eff*_ and B_11_, depending on magnetic layer thickness was determined based on the performed investigations of the above mentioned magnetic properties.

## Introduction

Magnetic thin films have been the subject of investigations for several decades, and up to now this area of research still remains very active. This fact is generally due to the proximity effects of surfaces and interfaces, making the thin films properties very different from their bulk counterparts.

Many actual thin film-based applications refer to spintronic devices based on half-metallic ferromagnetic materials with high spin polarization^1–5^. Using half-metallic electrodes with one of a conductor type with spin related channel of the density-of-states and another of an insulator or semiconductor type, theoretically up to 100% spin polarization at the Fermi level can be achieved^6,7^.

In this respect, due to low magnetic damping properties, among many candidates for half-metallic ferromagnetic electrodes^6,7^ full-Heusler materials occupy one of the leading position^8–11^. Using a general X_2_YZ chemical formula, where X and Y denote transition metals while Z denotes one of the main-group elements, most of half-metallic alloys belong to wide Co_2_YZ group^4,12^ with half-metallicity and high Curie temperature^4,13^. Co_2_YZ Heusler alloys are widely used in various spintronic applications, like tunnel magnetoresistance devices^14,15^, current-perpendicular-to-plane giant magnetoresistance devices^16–28^, lateral spin-valves^29–31^ and spin injectors into semiconductors^9,32,33^. The last studies also revealed topological semimetal properties in such materials^34^. Up to now Co_2_YZ Heusler alloys remain among the ones of greatest research interest^6,10,31,34–46^.

Among Co_2_YZ Heusler materials a special place is occupied by the quaternary alloys Co_2_Fe_0.4_Mn_0.6_Si (CFMS) and Co_2_FeGa_0.5_Ge_0.5_ (CFGG). The properties of CFMS alloys have been intensively investigated since 2006, when it was reported that an alloy of such composition possesses a more stable spin polarized band structure in comparison with other Co_2_YZ Heusler alloys^47,48^. Since that time CFMS alloys have been used to achieve large magnetoresistance effect^19,20^, tunnel magnetoresistance effect^49–52^, and to design a spin torque oscillators^53–56^. Highly spin polarized CFGG alloys were also investigated from the magnetoresistance point of view^18,21,29^. Novel graphene/CFGG heterostructures were recently reported to be used in high-performance graphene based spintronic devices^10^. Such heterostructures possess several significant advantages over the other reported ones^18,21,29^.

Magnetic damping properties of CFMS and CFGG alloys were reported to have low values of the Gilbert damping parameter — about 10^–3^ (Ref.^44,57–60^). The importance of a non-Gilbert-damping mechanism in such Heusler materials was studied in the paper^61^, indicating particular importance of the intrinsic two-magnon scattering mechanism. Strong perpendicular magnetic anisotropy was recently observed in ultrathin (below 2 nm) Co_2_YZ Heusler thin-film structures^62–65^, what was explained by the formation of a CoFe ordered alloy at the interface, which also caused an increase of the Gilbert damping parameter.

However, magnetoelastic properties of Co_2_YZ Heusler films remain poorly investigated, in spite of numerous studies on their magnetic properties performed during the last two decades. One of the main reasons for such situation is a limited number of methods, which allow studying magnetoelastic properties in thin films. The magnetoelastic part of magnetic anisotropy in Co_2_FeAl films was studied by Belmeguenai et al.^66,67^. The effects connected with piezoelectric strain were studied in Co_2_FeAl^68^ and Co_2_MnAl^69^. The changes in magnetic anisotropy associated with strain-induced tetragonal lattice distortion in Co_2_MnSi and Co_2_FeSi thin films were investigated by Pandey et al.^70^, nevertheless, this approach neglected the presence of surface effects.

Application of the magnetic films in spintronics at high frequencies requires low magnetic damping, but an increase of magnetoelastic effect usually leads to an increase of magnetic damping, for this reason, materials characterized by weak magnetoelastic effect are usually applied in spintronics.

However, there is a certain group of applications in which both low damping and high magnetostriction are required. As an example, the so-called acoustic spintronics can be given, in which spin currents are generated by mechanical excitation^71–73^.

Recently, the concept of the organic magnetoelastic coupling was presented^74^ leading to possibility of the rapid and effective mechanical control of spin polarization in organic multiferroic-magnetoelastic materials. To realize magnetoelastic coupling in organic crystals, a charge transfer-induced energy level splitting leading to spin polarization, and a strong spin–lattice coupling should occur. Charge transfer-induced energy level splitting leading to the room temperature ferromagnetism, and a significant magnetoelastic effect, was recently discovered in coronene-TCNQ^74^.

In non-reciprocal microwave devices, the magnetic properties can be controlled by means of voltage using the piezoelectric effect^75^. These devices could be improved if materials possessing low magnetic losses but a strong magnetic response to mechanical strain are available.

Recently magnetically soft epitaxial spinel NiZnAl-ferrite thin films were found, which possess a low magnetic damping and strong magnetoelastic coupling at the same time, revealing a new class of low-loss magnetoelastic thin film materials^76^.

In spite of the several experimental studies reported so far^77,78^, the mechanism of a correlation between magnetoelastic and magnetic damping properties in thin film materials is not sufficiently understood yet. Another important factor is that most studies related to such correlations were reported only for the permalloy type materials^77–79^.

In this work we report studies of magnetoelastic and magnetic damping properties of epitaxial CFMS and CFGG thin films^80^ with an additional Ag buffer layer and different magnetic layer thicknesses. Magnetoelastic effect was studied by means of the Strain Modulated Ferromagnetic Resonance (FMR) technique^81,82^, which enables the determination of magnetoelastic tensor components of thin film materials by applying a controlled strain to a metallic thin film. Special emphasis was placed on the correlation between magnetoelastic constants B_11_ and the effective magnetic damping parameter α_*eff*_ for magnetic layers of a constant composition but different thickness.

## Experimental

**Figure 1:**
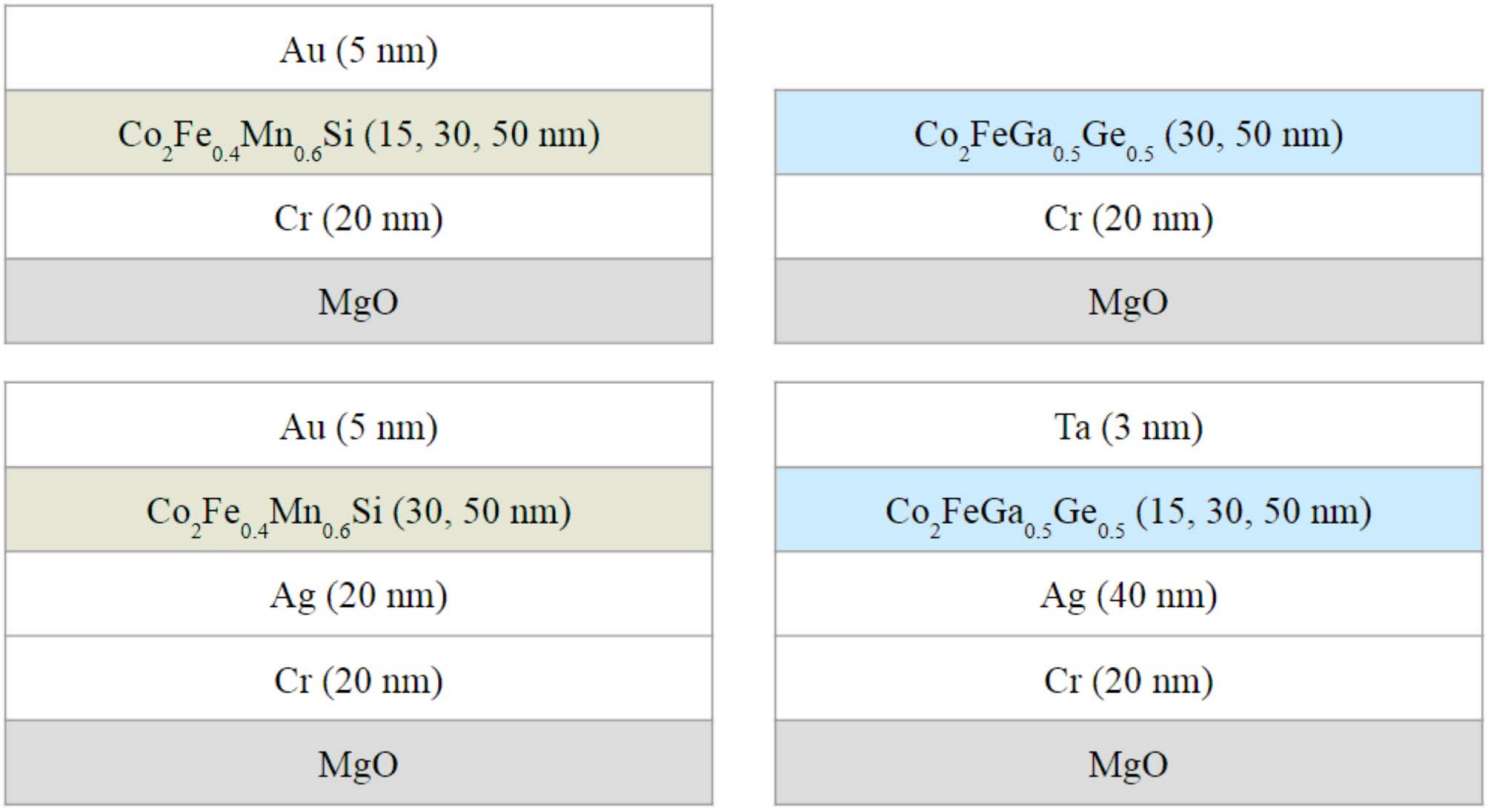
Investigated samples belong to 4 series: CFMS and CFGG on Cr buffer layer with or without the additional Ag buffer layer.

Four series of Heusler alloy Co_2_YZ thin films were grown on MgO (001) substrates (see Fig. 1) with the Cr buffer layer of 20-nm-thickness using UHV-compatible magnetron sputtering systems with previously calibrated alloy targets. The deposition rate for each sputtering target was obtained by measuring the thickness of the single layer sample prepared with a fixed sputtering input power. The Cr buffer layer was applied in order to decrease the lattice mismatch between the Heusler alloy magnetic layer and the MgO substrate, and thus enable epitaxial growth. Two different sputtering systems were used for the CFMS and CFGG samples: multi-cathode sputtering machine (ULVAC Inc.) for CFMS and single-cathode sputtering machine with revolver-type target changer (Eiko Corp.) for CFGG. For both sputtering systems, the base pressure was of the order of 10^–7^–10^–8^ Pa, and Ar gas pressure was in the range about 0.1–1 Pa. Before starting the deposition, the annealing of MgO substrate at 600 °C was carried out. The 20-nm-thick Cr buffer layers were deposited at room temperature and in situ annealed at 600 °C to obtain a low roughness surface. Apart from Co_2_YZ samples deposited on the Cr buffer, the samples with an additional Ag buffer layer on the Cr one were grown with the aim of reducing of Cr atoms diffusion into a magnetic layer. Most of the films were additionally covered by a 5 nm Au or 3 nm Ta capping layer in order to prevent surface oxidation. Magnetic layers were deposited at room temperature (except for CFGG with Ag buffer samples which were grown at 250 °C) and were subsequently annealed at 500 °C for 20 min in order to promote the chemical ordering^35,83^. In situ reflection high-energy electron diffraction (RHEED) images after each layer deposition process were recorded and ex situ X-ray diffraction (XRD) measurements were performed to control the surface and structure quality of grown thin films. Series of the films with different magnetic layer thicknesses (from 15 to 50 nm) were grown in order to check how the thickness of the magnetic layer influence the quality of the epitaxial growth and structural ordering. Studying of the samples series with different magnetic layer thicknesses enables determination of the surface effects (surface anisotropy and surface magnetoelastic coupling) influence on magnetic, magnetoelastic and damping properties of the investigated samples.

To determine magnetoelastic constants of the magnetic thin films, Strain Modulated FMR technique was applied, which uses a standard X-band spectrometer equipped with an additional system for applying the controlled strain modulation (all details are given in Ref.^82^). The observed FMR line shift due to the strain effect contains information on the magnetoelastic tensor B_ijkl_ components. In this case magnetic free energy of the system includes also a magnetoelastic energy contribution.

1 $$E_{ME} = \sum\limits_{i,j,k,l = 1}^{3} {B_{ijkl} \alpha_{i} \alpha_{j} \varepsilon_{kl} }$$

where α_i_ and α_j_ are directional cosines of the magnetization vector, ε_kl_ denotes the strain tensor. For an isotropic case the tensor B_ijkl_ can be constructed using only one magnetoelastic constant, e.g. B_1111_; in Voigt notation B_1111_ = B_11_.

The thin film sample face was glued to the quartz rod, with square (3 × 3 mm^3^) cross-section in which periodic strains were induced by a piezoelectric generator^82^. The strains induced in the sample plane were ε_11_ and ε_22_, in the direction parallel and perpendicular to the rod, respectively. In our system no shear strain in the plane of the film was induced (i.e. ε_12_ = 0). The crystallographic [110] axis of the epitaxially grown Heusler alloy layer was oriented parallel to the rod, and the external magnetic field in Strain Modulated FMR experiments was oriented perpendicular to the rod and parallel to the investigated film plane. From the shift of a FMR line induced by the strain the magnetoelastic constant B_11_ was calculated assuming the magnetoelastic properties of the magnetic layer are isotropic.

For most of the studied samples Agilent Technologies Vector Network Analyzer FMR spectrometer with a micro-stripe line was used to record FMR curves either with a frequency swept at a fixed magnetic field strength or with magnetic field swept at a fixed frequency, using the measurement protocol given in^84^.

In all Vector Network Analyzer FMR experiments the external magnetic field was applied parallel to the film plane, and parallel to [110] crystallographic axis of the epitaxially grown magnetic layer. For this reason, the dragging effect, which occurs when the sample magnetization is not parallel to the external magnetic field, could be neglected in the analysis of frequency dependence of the FMR linewidth^85^. Bruker EMX X-band spectrometer with a resonant cavity was used for the basic FMR measurements. A SQUID magnetometer was used to determine the saturation magnetization of all samples. In order to check the crystallographic quality of the layer and possible deformation of its crystal lattice, a high-resolution X-ray diffractometer with a 4-reflection Ge (220) monochromator and X-ray mirror, radiation — Cu_Kα1_ and an analyzer in front of a proportional detector was used for the sample with the thickest CFMS layer (50 nm) grown on the Ag buffer.

## Results and discussion

**Figure 2:**
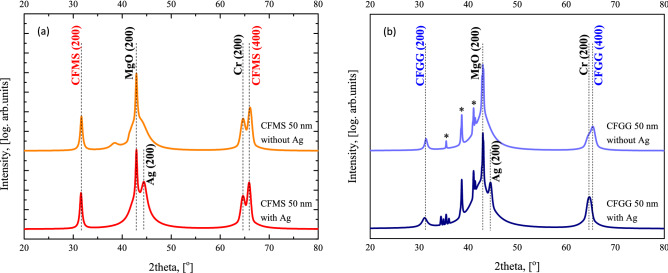
XRD scan patterns for CFMS (**a**) and CFGG (**b**) samples with 50-nm-thick magnetic layer and with/without the Ag buffer layer; the peaks indicated by *originate from the MgO single crystal substrate.

The RHEED patterns obtained for the magnetic Heusler layers revealed a low roughness surface and the epitaxial growth with relationships of crystallographic directions: MgO (001) || Cr (001) || magnetic layer (001) or MgO (001) || Cr (001) || Ag (001) || magnetic layer (001) and for all the samples superlattice (200) streaks were clearly observed. In accordance with the RHEED patterns, the (200) superlattice diffractions as well as the (400) fundamental diffractions of the Heusler layers were clearly observed in XRD patterns for all the samples (see Fig. 2). The well-defined (400) diffraction peak indicates that the samples are well crystallized, having cubic symmetry. The intense (200) superlattice peak indicates that all magnetic layers have at least the B2 ordered structure.

**Figure 3:**
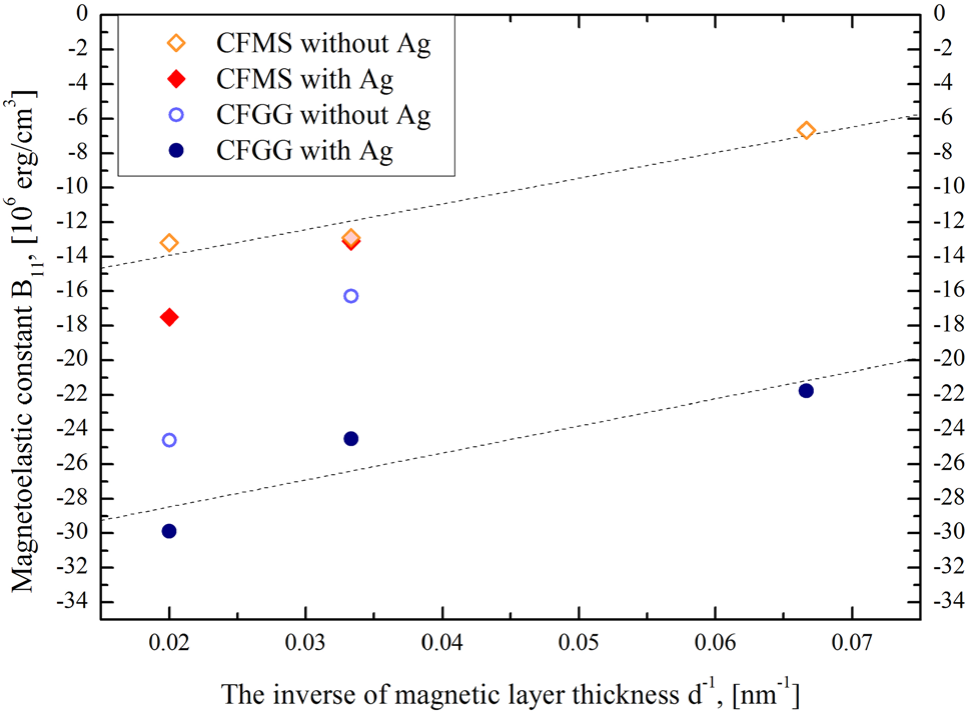
Magnetoelastic constant B_11_ for CFMS and CFGG films as a function of the inverse of magnetic layer thickness at room temperature.

Magnetoelastic interactions in the studied samples are relatively weak (part of the data was previously published^80^). Experimentally determined magnetoelastic constants B_11_ have values in the range of − (6–30) × 10^6^ erg/cm^3^ (see Table 1), which are close to the values reported for Co_2_YSi (with Y = Fe or Mn)^70^ and for Co_2_FeAl^66^. Figure 3 illustrates the magnetoelastic constants dependence on the film thickness. The saturation magnetostriction λ_S_ can be described as:

2 $$\lambda_{S} = - \frac{{B_{11} }}{{c_{11} - c_{12} }}$$

**Table 1:** The values of the magnetoelastic tensor components B_11_ and corresponding saturation magnetostriction λ_S_ obtained for CFMS and CFGG thin film samples.

d, nm	Co_2_Fe_0.4_Mn_0.6_Si				Co_2_FeGa_0.5_Ge_0.5_	
	With Ag		Without Ag		With Ag	Without Ag
	B_11_, 10^6^ erg/cm^3^	λ_S_, 10^–5^	B_11_, 10^6^ erg/cm^3^	λ_S_, 10^–5^	B_11_, 10^6^ erg/cm^3^	B_11_, 10^6^ erg/cm^3^
15	–	–	− 6.67	0.54	− 21.74	–
30	− 13.10	1.08	− 12.90	1.06	− 24.52	− 16.30
50	− 17.50	1.44	− 13.20	1.08	− 29.86	− 24.60

Elastic constants used for λ_S_ calculation are taken from^87^.

where c_11_ and c_12_ are elastic constants. The values of λ_S_ for all the studied samples are positive and similar to the values obtained for Co_2_FeAl^66^ and Co_2_MnAl^86^ Heusler alloy thin films. For all 4 series of the studied samples an increase of the magnetoelastic constant absolute value and saturation magnetostriction with increasing of the magnetic layer thickness was observed.

Similar to the concept of the surface anisotropy^88^, the concept of the so-called surface magnetoelastic coupling was introduced^89,90^. In this case the magnetoelastic constant can be, formally, divided into two parts:

3 $$B_{11} = B_{11,V} + \frac{{2B_{11,S} }}{d},$$

where *d* is a magnetic layer thickness, B_11,V_ and B_11,S_ are the volume and surface components of the magnetoelastic constant, respectively. Using the experimental data of the thickness dependence of magnetoelastic constants B_11_, shown in Fig. 3, and assuming a linear fit (see dotted lines in Fig. 3) the B_11,V_ and B_11,S_ parameters values were determined for the samples series, containing 3 samples: for the CFMS thin films without the additional Ag buffer layer: B_11,V_(CFMS) = − 17.0 × 10^6^ erg/cm^3^, B_11,S_(CFMS) = 7.5 erg/cm^2^, and for the Ag-buffered CFGG thin films: B_11,V_(CFGG) = − 31.6 × 10^6^ erg/cm^3^, B_11,S_(CFGG) = 7.8 erg/cm^2^.

Very little experimental work was up to now devoted to the study of surface magnetoelastic coupling. The estimated by us surface components of the magnetoelastic constants for CFMS and CFGG films are very close to those found in epitaxial iron films deposited on GsAs^91,92^. Although the volume components of the magnetoelastic constants in these iron films were found to depend on both sample preparation procedure and the structure of the films (using additional overlayers^91^), the surface components of all the investigated films were very similar: b_1_^S^ ~ 11 erg/cm^3^. For an isotropic sample b_1_^S^ = (3/2)B_11,S_, which gives a good consistency with our results.

For all investigated in this study series of samples the magnetoelastic constant absolute values decrease with decreasing magnetic layer thickness (see Fig. 3). Hence, the magnetoelastic effect in such thin layers is weaker than that expected in bulk materials.

The obtained for CFMS samples saturation magnetostriction value λ_S_ = 1.44 × 10^–5^, is in accordance with the experimental value reported for Co_2_Cr_0.6_Fe_0.4_Al Heusler alloy bulk sample^93^ and the recent theoretical calculations for Co_2_XAl Heusler alloys^94^, where X = V, Ti, Cr, Mn, Fe.

The concept of surface magnetoelastic coupling^89,90^ can explain the incerase of the magnetoelastic constants absolute values with increasing thickness for the studied magnetic layers. Existing theoretical papers predict several mechanisms responsible for surface magnetoelastic coupling (see e.g. review paper^95^). Among them the most important are spin–orbit interactions (single-ion model) and dipole–dipole interactions. Other mechanisms observed in both single layers and multilayers are non-linear contributions to bulk magnetoelastic coefficients due to surface strains and surface roughness effects. Also the presence of interdiffusion layers which are formed at the interface should be taken into account.

As the magnetoelastic constant can be changed by a strain, their changes with the thickness of the magnetic layers may be also a result of structural relaxation. In our previous studies^80^, we found the magnitude of the perpendicular magnetocrystalline anisotropy of investigated samples to be decreasing with increasing magnetic layer thickness. Such a behavior could suggest an occurrence of structural relaxation. For the strained Heusler alloys their cubic symmetry will be lowered and in order to check such a possibility additional X-ray studies were performed.

**Figure 4:**
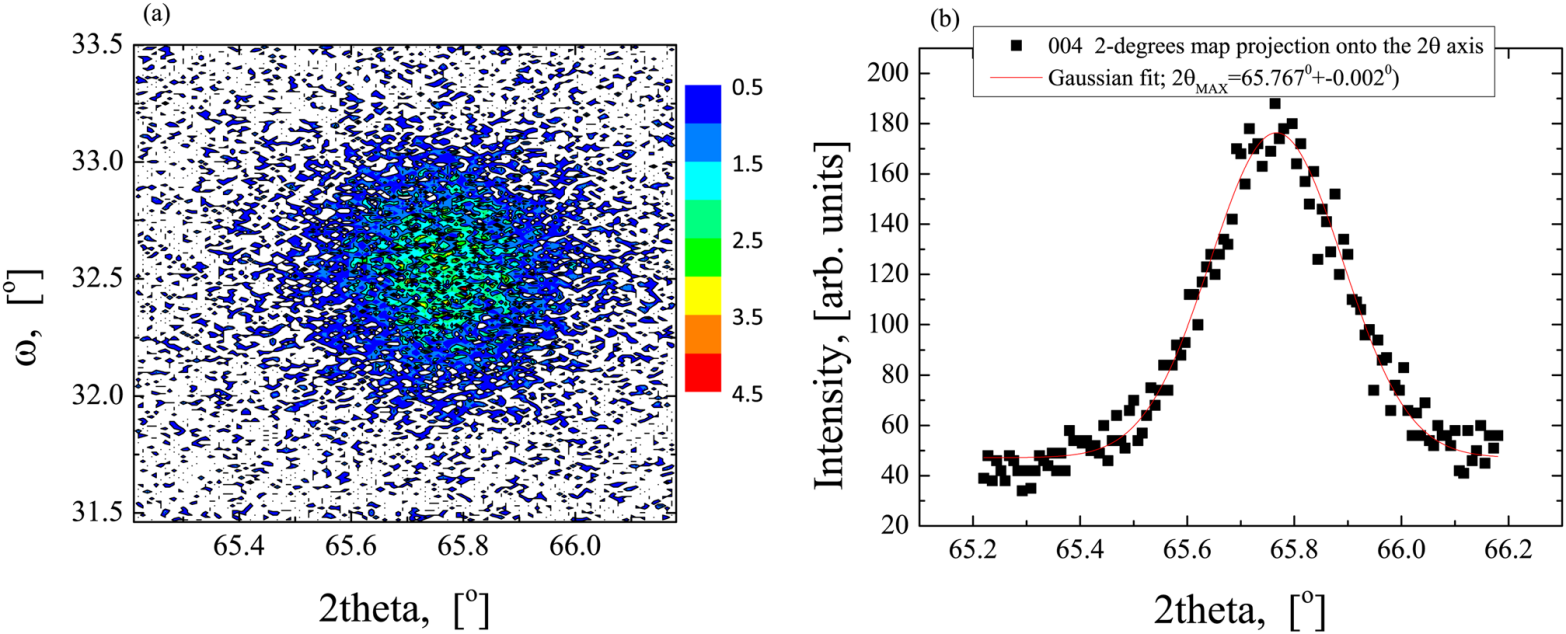
The X-ray intensity distribution around the (004) reflection (**a**) and nod projection into the 2θ axis (**b**) for the 50-nm-thick CFMS layer grown on the Ag buffer.

**Figure 5:**
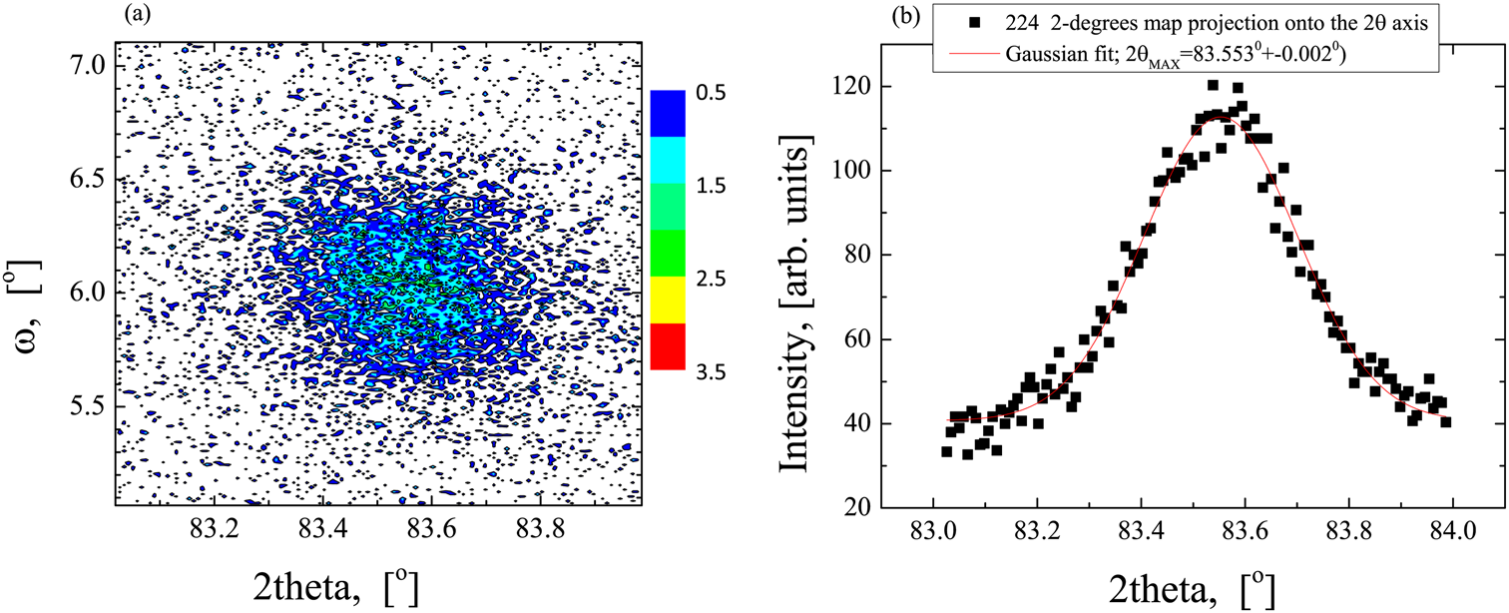
The X-ray intensity distribution around the (224) reflection (**a**) and nod projection into the 2θ axis (**b**) for the 50-nm-thick CFMS layer grown on the Ag buffer.

For the sample with the thickest CFMS layer (50 nm) grown on the Ag buffer additional detailed XRD studies were performed (see Figs. 4 and 5). From the rocking curve measurements of the (004) and (224) reflections, we obtained the half-widths of the ω-scans equal to 0.75° and 0.65°, respectively. This indicates that, in addition to the undoubted widening associated with a small layer thickness, we are probably observing the influence of mosaic-type defects. In order to calculate the lattice deformation, 2-dimensional X-ray diffusion scattering maps around the symmetric (004) (Fig. 4a) and asymmetric (224) (Fig. 5a) reflexes were obtained. Projections of the map nodes on the 2θ axis and fitting Gaussian curves to the obtained measurement points allowed to determine the interplanar distances for (004) and (224) planes (see Figs. 4b and 5b, respectively). Hence, we obtained lattice unit parameters: a_⊥_ = 5.675 ± 0.001 Å and a_||_= 5.643 ± 0.005 Å indicating a tetragonal deformation of about 0.5%, which results in the appearance of the strain induced anisotropy. Errors in determining lattice unit parameters were assessed from the ambiguity of fitting Gaussian curves to projections of maps and measurement method.

As the tetragonal distortion of the investigated sample was found to be very small, it seems that not the structural relaxation but rather the surface magnetoelastic coupling is responsible for the observed magnetoelastic constants absolute values increase with increasing thickness of the magnetic layer. It is possible that the observed changes of the magnetoelastic constants may be also connected with the thickness dependent structural ordering occurring for thicker layers as it was reported for Co_2_FeSi layers^96^.

Using the formula for magnetoelastic energy (1) for an isotropic sample case and assuming the tetragonal distortion (ε_11_ = ε_22_) the formula for the strain induced contribution to the uniaxial anisotropy constant K_SI_ can be written as:

4 $$K_{SI} = \frac{3}{2}B_{11} \left( {\varepsilon_{11} - \varepsilon_{33} } \right),$$

where the strain component ε_33_ = − 2(c_12_/c_11_)ε_11_.

Taking the data from our experiments: ε_11_ = − 2.44 × 10^–3^, B_11_ = − 17.50 × 10^6^ and elastic constants^87^, we have obtained the value of K_SI_ = (1.39 ± 0.23) × 10^5^ erg/cm^3^. It has the opposite sign and a small value in comparison to the overall magnetocrystalline anisotropy of relatively low and negative value |K|< 1.5 × 10^6^ erg/cm^3^ which was determined from FMR/SQUID studies^80^. Strain causes an increase of the anisotropy constant and reduces its absolute value. The minimal tetragonal distortion value, which is necessary to switch the magnetic layer anisotropy from an easy-plane to an easy axis type, was estimated to be at least ε_11-min_ ≈ − 0.07; but such a large strain is not likely to be present in our samples.

Such a large estimated critical value of the distortion results from the fact that a large demagnetizing energy in a thin film (proportional to the magnetization) must be overcome by the strain induced anisotropy, which is proportional to a (relatively small) magnetoelastic constant.

Magnetic damping is proportional to the FMR linewidth. However there are many mechanisms that may be responsible for the dissipation of magnetic energy in thin magnetic films, thus the full width at half maximum in registered FMR spectra can include the following contributions^85^: ΔH = ΔH_0_ + ΔH^TMS^ + ΔH^G+sp^, where ΔH_0_ denotes the frequency-independent sample inhomogeneity contribution, ΔH^TMS^ denotes the two-magnon scattering contribution (TMS), while the last term ΔH^G+sp^ contains the spin pumping (SP) and Gilbert damping contributions and is proportional to α_*eff*_ × *f*, where α_*eff*_ is an effective magnetic damping parameter and *f* is the FMR frequency^85^. Considering the TMS mechanism is especially important for the samples characterized by low Gilbert damping when the external magnetic field is parallel to the film^97^.

**Figure 6:**
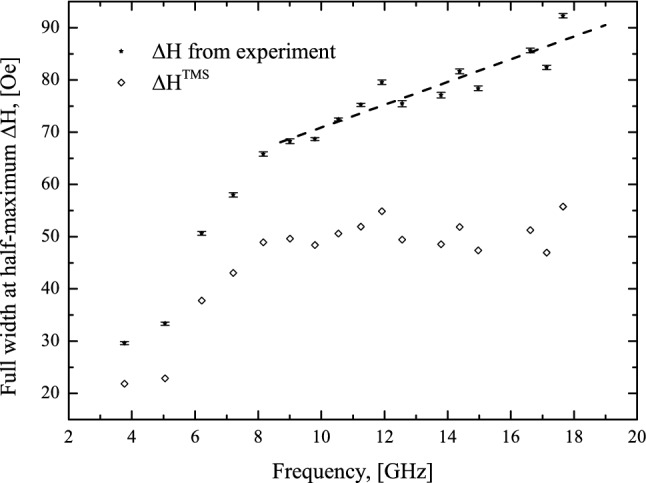
Resonance line full width at half maximum for the 30 nm CFMS sample without additional silver buffer layer as a function of frequency at room temperature. Solid line presents a linear fit for the frequencies above 8 GHz, which slope is proportional to α_*eff*_. Empty symbols present the extracted as described above, two magnon scattering contribution (TMS).

Figure 6 shows the experimental frequency dependence of half-maximum width ∆H for the 30 nm CFMS sample without additional silver buffer layer. It is clearly seen that at frequencies below 8 GHz this dependence is nonlinear and a slope of the curve decreases with increasing frequency but above 8 GHz, almost linear dependence was observed. Such a dependence of ∆H is typical if the TMS contributes to the damping processes^98,99^. In the low frequency range the ∆H^TMS^ vs. frequency is nonlinear, while it saturates at high frequencies. For this reason in the high frequency range, the slope of the ∆H can be assumed to be proportional to effective magnetic damping parameter α_***eff***_. The estimated values of α_*eff*_ for all samples studied in our experiments are presented in Tab.2. After subtraction of ΔH^G+sp^ and ΔH_0_ the ΔH^TMS^ can be evaluated, which is also presented in Fig. 6. It can be seen that the ΔH^TMS^ contribution to the total ∆H is relatively strong, which is typical for in-plane broad band FMR experiments if the effective magnetic damping parameter is relatively small^97^.

**Table 2:** The effective magnetic damping parameter α_*eff*_ values for the studied CFMS and CFGG samples.

d, nm	Co_2_Fe_0.4_Mn_0.6_Si		Co_2_FeGa_0.5_Ge_0.5_	
	With Ag	Without Ag	With Ag	Without Ag
	α_*eff*_, 10^–3^	α_*eff*_, 10^–3^	α_*eff*_, 10^–3^	α_*eff*_, 10^–3^
15	–	1.00 ± 0.82	5.97 ± 0.79	–
30	2.59 ± 0.74	1.90 ± 0.10	1.85 ± 0.70	7.20 ± 1.00
50	3.88 ± 0.89	4.50 ± 0.19	1.72 ± 0.14	11.94 ± 0.76

The magnetic damping parameters in the studied samples are evaluated to be of the order of 10^–3^; but the application of the additional Ag buffer layer in case of the CFGG samples resulted in a significant reduction of the α_*eff*_ value.

**Figure 7:**
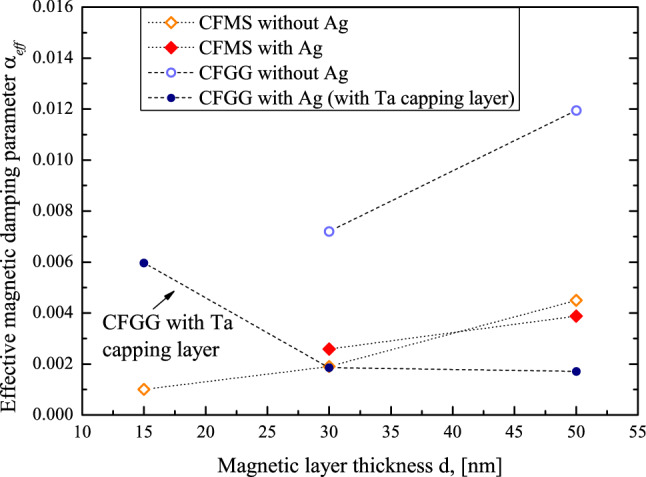
Effective magnetic damping parameter α_*eff*_ for CFMS and CFGG samples as a function of magnetic layer thickness at room temperature.

It should be noted that contrary to other samples, the Ag-buffered CFGG thin films have the cover layer of Ta, which has a strong spin–orbit coupling and as a consequence, the value of the frequency proportional contribution ΔH^G+sp^ ~ α_*eff*_ × *f* is increased due to the presence of the spin pumping phenomenon^100^ and only for these samples α_*eff*_ is decreasing with increasing magnetic layer thickness (see column 4 in Table 2). In the case of other investigated samples which were without Ta — but having Au cover layer—it was found that the effective magnetic damping parameter α_*eff*_ increases with the magnetic layer thickness (see Fig. 7).

**Figure 8:**
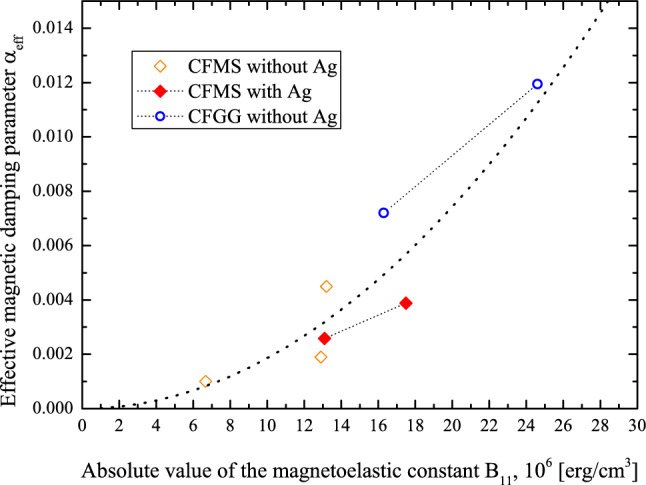
The correlation between the absolute values of the magnetoelastic constants B_11_, and the effective magnetic damping parameter α_*eff*_ for the three series of the samples, for which the influence of the spin pumping on the magnetic damping can be neglected. Dashed line shows the fit using quadratic function.

It should be emphasized that for all studied samples, the increase of magnetic layer thickness is accompanied by the increase of the magnetoelastic constant B_11_ absolute value. Therefore, for the samples, where effective magnetic damping parameter α_*eff*_ may be considered as Gilbert damping parameter α (without spin pumping contribution due to a presence of Ta), the increase of the absolute value of magnetoelastic constant is accompanied by the increase of the damping parameter (see also Fig. 8).

It is generally assumed^77,79,101,102^ that the amplification of the magnetoelastic effect should lead to an increase of magnetic damping. In the case of thin magnetic films, this hypothesis was verified by a number of experimental studies conducted mainly for Ni–Fe films. For example the correlation between magnetic damping and magnetostriction in permalloy films of different compositions was reported by several authors^77,79^. The variation of the magnetostriction constant from − 0.7 × 10^–5^ to 1.5 × 10^–5^ was caused by changing of a Ni-to-Fe ratio where λ = 0 for Ni_80_Fe_20_ composition. Nevertheless, it is difficult to find formulas which correlate the magnetoelastic constants and damping parameters quantitatively. Bonin et al.^79^ assumed that the correlation of magnetic damping with strain fluctuations was induced by the magnetoelastic effect. To describe the fluctuation processes that affect damping, originating during the magnetization reorientation process, the use of nonequilibrium statistical mechanics is required. In Ref.^79^, the self-organized criticality^103^ was used to perform the experimental data analysis. A model of correlation between the magnetostriction and relaxation mechanism was also presented in Ref.^101^, where the coupling between the magnetic motion and lattice was based purely on continuum arguments concerning magnetostriction. In Ref.^102^, dynamics of magnetization coupled to a thermal bath of elastic modes was analyzed. Both models, in Refs.^101^ and^102^, predict a damping factor to be proportional to the square of the magnetoelastic constants. Most importantly in Ref.^102^, an increase of the damping factor with the increase of the magnetic layer thickness was anticipated what is confirmed by our results.

Figure 8 shows the correlation between the absolute values of the magnetoelastic constants B_11_, and the effective magnetic damping parameter α_*eff*_ for the three series of the samples studied in our experiments, for which the influence of the spin pumping on the magnetic damping can be neglected (no Ta capping layer). Experimental data of all three series were collected in one plot. Although the scatter of experimental data is large, one can see a general tendency of the effective damping increasing with an increase of B_11_ magnitude. In Fig. 8 a fit using the quadratic function is also shown. As discussed above, such dependence was predicted by some of theoretical models describing correlations between magnetoelastic properties and damping^101,102^.

However, it must be kept in mind that beside the magnetoelastic effect also other parameters influence the magnetic damping, and if these parameters change with the thickness of the magnetic layer this can also result in the changes of magnetic damping. In particular, half-metallicity and Gilbert damping factor in Co_2_Fe_x_Mn_1-x_Si Heusler alloys depending on the film composition were investigated in Ref.^57^ and Gilbert damping parameter in Co_2_Fe_x_Mn_1-x_Si Heusler alloys was shown to be correlated with their band structure The minimum of the damping parameter (α ≈ 0.003) was found for the composition with x = 0.4. The results were discussed assuming the Gilbert damping constant to be proportional to the square of the spin–orbit coupling parameter and total density of states of the d-band at Fermi energy.

In the experimental studies performed earlier by other researches^77,79^, the variation of magnetoelastic properties was induced by the change of film composition. In the case of the results obtained and discussed in this paper, the samples have a constant composition in one series of samples and an increase of magnetic damping is correlated with an increase of the absolute value of magnetoelastic constants, which increases with increasing magnetic layer thickness.

Such an effect has not been observed so far. The observation and study of such correlations are important also from the point of view of applications in spin-mechanical devices, for which thin film materials possessing low magnetic damping with a sizable magnetoelastic effect are sought^76^.

Although the correlation between the magnetoelastic properties and damping factor in thin films is not fully understood yet, most models developed up to date predict an increase of the damping factor with the increase of the magnetoelastic constants magnitude and/or with the thickness of a magnetic layer. This is consistent with our experimental observations. Because of the lack of several important parameters included in the existing theoretical models like the elastic relaxation time or the exchange stiffness constant, quantitative comparison of our results with these models is difficult. It should also be remembered that the magnetoelastic mechanism may not be the only one responsible for the changes of the damping coefficient values observed in our experiments, e.g. the conductivity of the samples was neglected in our considerations. The changes of the structural ordering with the thickness of magnetic layer may also influence the band structure, and thus the Gilbert damping.

## Conclusions

Magnetoelastic properties and magnetic damping for several series of quaternary Co_2_Fe_0.4_Mn_0.6_Si and Co_2_FeGa_0.5_Ge_0.5_ Heusler alloy thin magnetic films were determined by the Strain Modulated FMR and the Vector Network Analyzer FMR methods, respectively. The magnetoelastic constants were found to have relatively small and negative values while saturation magnetostriction for all the studied samples was positive. It was shown that two-magnon scattering and spin pumping phenomena play an important role in the magnetic damping of microwaves in the studied magnetic thin films.

The frequency dependent FMR linewidths were found to be strongly inhomogeneous; the resonance line FWHM nonlinear dependence evidences that the inhomogeneous broadening is correlated mainly with two magnon scattering processes. It was revealed that the magnetic damping in the investigated samples is strongly influenced by the type of metal used for a buffer or cover layer; where specifically the use of Ta cover layer leads to a strong spin pumping phenomenon appearance. Linear dependence of the resonance line at high frequencies enabled determination of the magnetic Gilbert damping parameter for the studied samples, assuming that the TMS contribution at these frequencies is saturated.

The saturation magnetostriction (λ ≈ 10^–5^) and low magnetic damping parameter (α_*eff*_ ≈ 10^–3^) values in the investigated Heusler alloys thin films are similar to the results of the pioneering work^73^, where a new class of materials promising for spin-mechanical devices was reported. Calculated strain, which is necessary to switch the magnetic anisotropy from the easy-plane to the magnetization easy axis type, is too large (ε_11-min_ ≈ − 0.07) to be achieved in the epitaxially grown magnetic layers.

In the samples for which it was possible to neglect the spin pumping phenomenon (i.e. without the Ta cover layer), the correlation between the Gilbert damping parameter and magnetoelastic constants was obtained. Based on the fact that both the magnetic damping parameter and the absolute value of the magnetoelastic constant increase with magnetic layer thickness, it was concluded that the enhanced magnetoelastic effects are accompanied by a stronger magnetic damping. An increase of the absolute values of magnetoelastic constants with increasing thickness of the magnetic layer can be explained as a result of surface magnetoelastic coupling and/or the thickness dependent structural ordering. An increase of Gilbert damping can be correlated with increasing magnetoelastic constants or changes in the band structure caused by the changes of structural ordering.

## Data Availability

All data reported in this manuscript is available from the corresponding author on a reasonable request.
